# Supplementary material for: Mesothelial Cells Exhibit Characteristics of Perivascular Cells in an In Vitro Angiogenesis Assay
Source: Cells. 2023 Oct 11;12(20):2436. doi: 10.3390/cells12202436 (PMC10605208; doi:10.3390/cells12202436)
Supplement: Supplementary file 1 [file cells-12-02436-s001.zip › cells-2581165-supplementary.pdf]

Article

Mesothelial Cells Exhibit Characteristics of Perivascular Cells in an In Vitro Angiogenesis Assay

Chrysa Koukorava [1$^], Kelly Ward [2$], Katie Ahmed [1], Shrouq Almaghrabi [1], Sumaya Dauleh [2], Sofia M Pereira [2], Arthur Taylor [2,3], Malcolm Haddrick [3], Michael J Cross [1,*] and Bettina Wilm [2,4,*]

Supplementary material

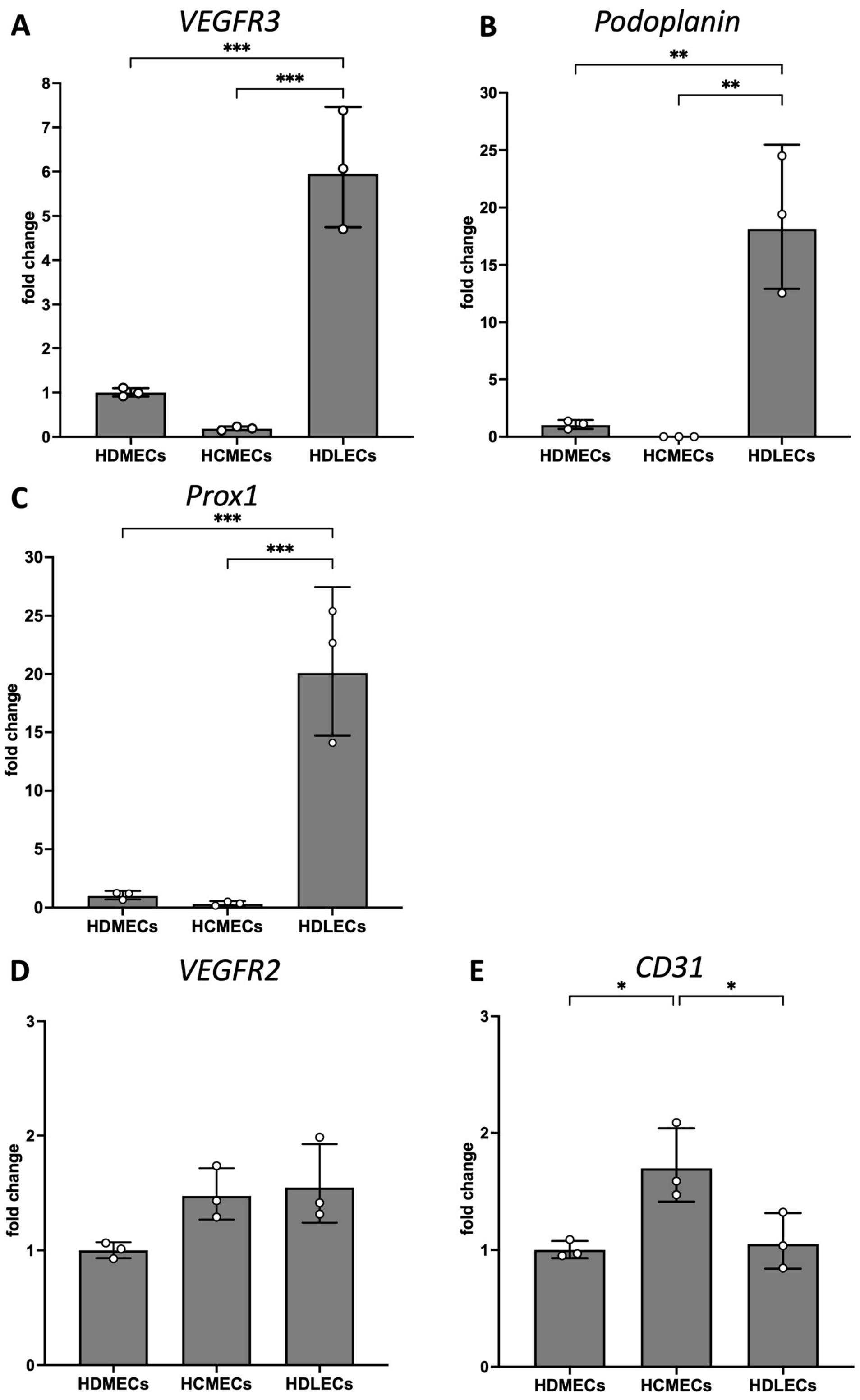

Supplementary Figure S1. Authentication of vascular endothelial cells. Total RNA was extracted from human dermal microvascular endothelial cells (HDMECs), human cardiac microvascular endothelial cells (HDMECs) and human dermal lymphatic endothelial cells (HDLECs). The expression levels of *VEGFR3*, *PODOPLANIN* and *PROX-1* (lymphatic endothelial specific genes, **A**-**C**) and *CD31* and *VEGFR2* (endothelial specific genes, **D**, **E**) were analysed by qRT-PCR. Data displayed as Geometric mean ± geometric SD, one-way ANOVA followed by Tukey's post-hoc test where significance was defined by a *p*-value ≤ 0.05. *p*-value≤0.05 (*), ≤ 0.005 (**), < 0.001 (***).

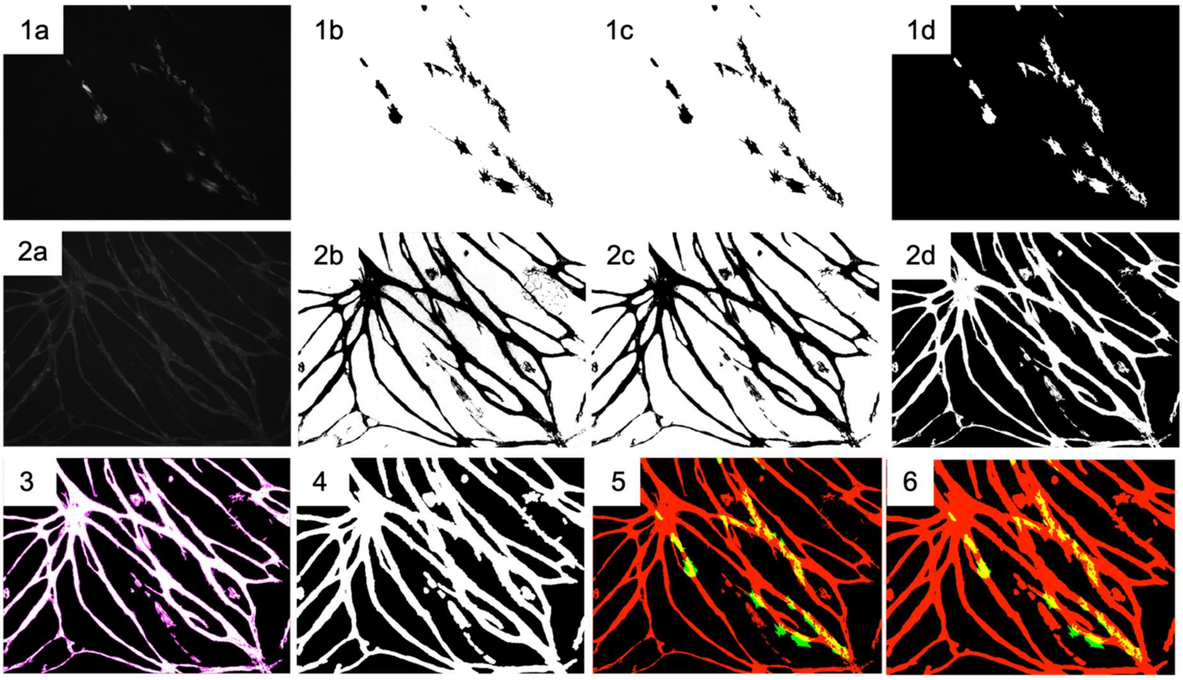

Supplementary Figure S2. Representative images demonstrating quantification of GFP-labelled cells to endothelial tubes. After splitting the image channels for the GFP-labelled cells (**1a**) and endothelial cells stained with CD31 (**2a**), the images are thresholded (**1b**,**2b**), removed of any background (**1c**,**2c**), and lastly inverted (**1d**,**2d**). The area that the endothelial tubes occupy is selected (**3**) and expanded by 5 μm in each direction, with the expanded area being filled in (**4**). Composite images are created of the area the tubes occupy in relation to the GFP signal pre- (**5**) and post-enlargement (**6**). All images are 10x. Scale bar = 200 μm.

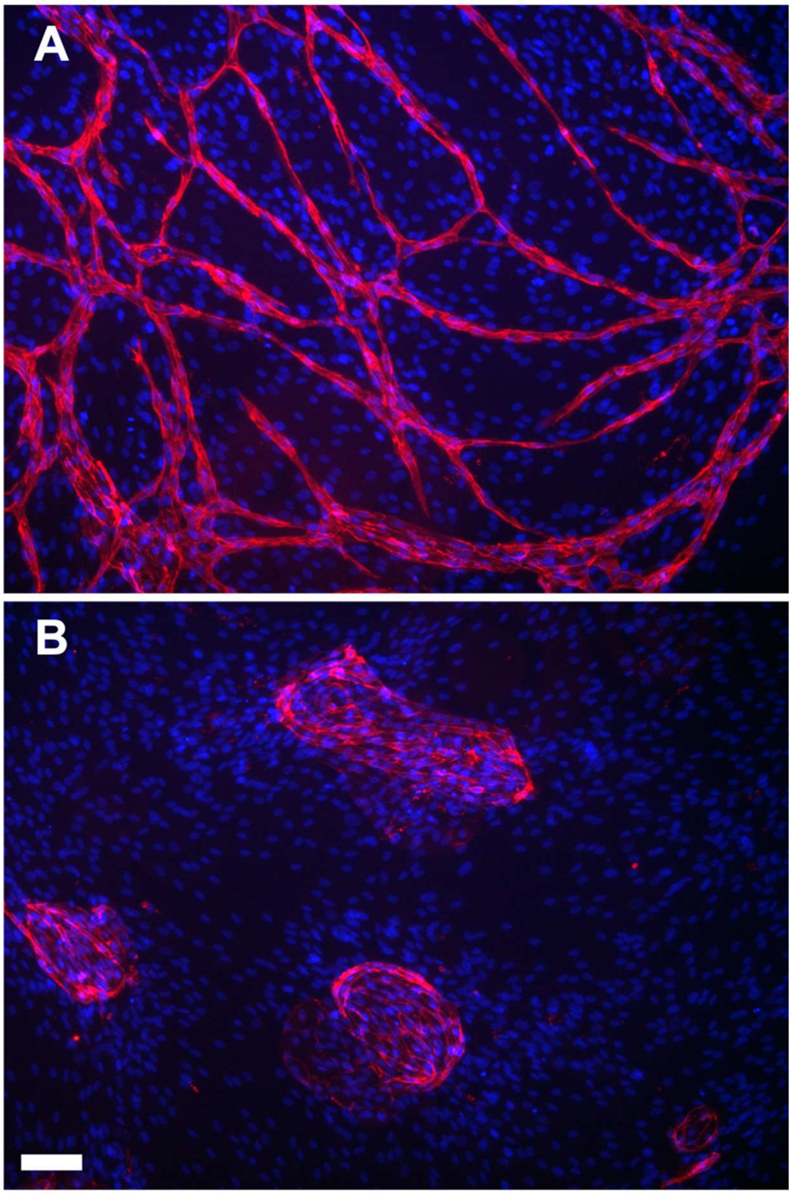

Supplementary Figure S3. Co-culture of HDMECs with NHDFs, in presence (**A**) or absence (**B**) of 50 ng/ml VEGF-A_{165}. Fixed cells were immunolabelled with CD31 (red), and nuclei visualized with DAPI. Scale bars are 100 µm.

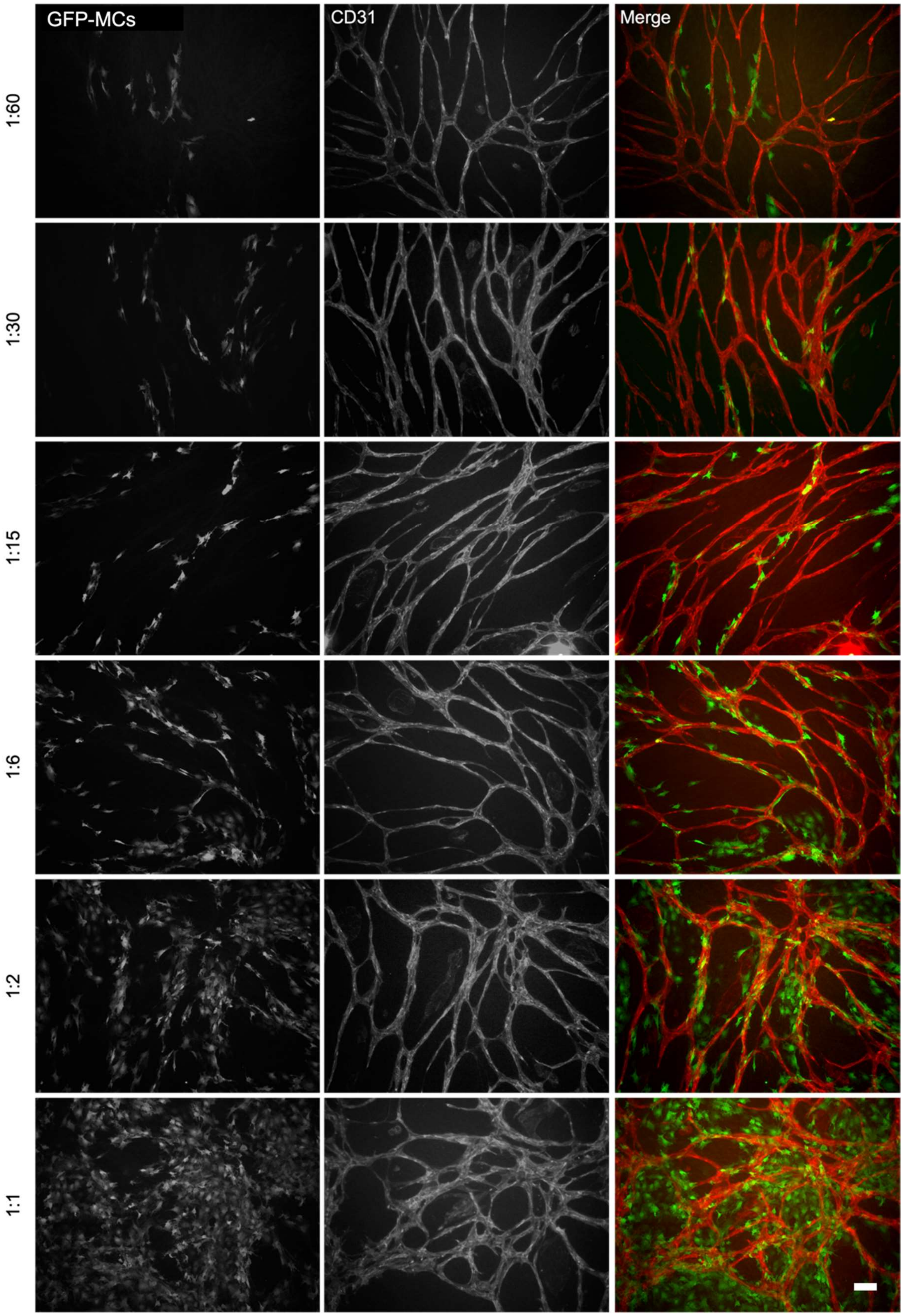

Supplementary Figure S4. Representative images of immunofluorescence staining for GFP (GFP-MCs) and CD31 showing specific GFP-MCs alignment to HDMECs at different seeding ratios in the vitro angiogenesis experiment in the presence of VEGF-A_{165}. GFP-MCs had been added into the assay at the ratios indicated (ratios shown as number of GFP-MCs : number of HDMECs). Scale bar = 100 μm.

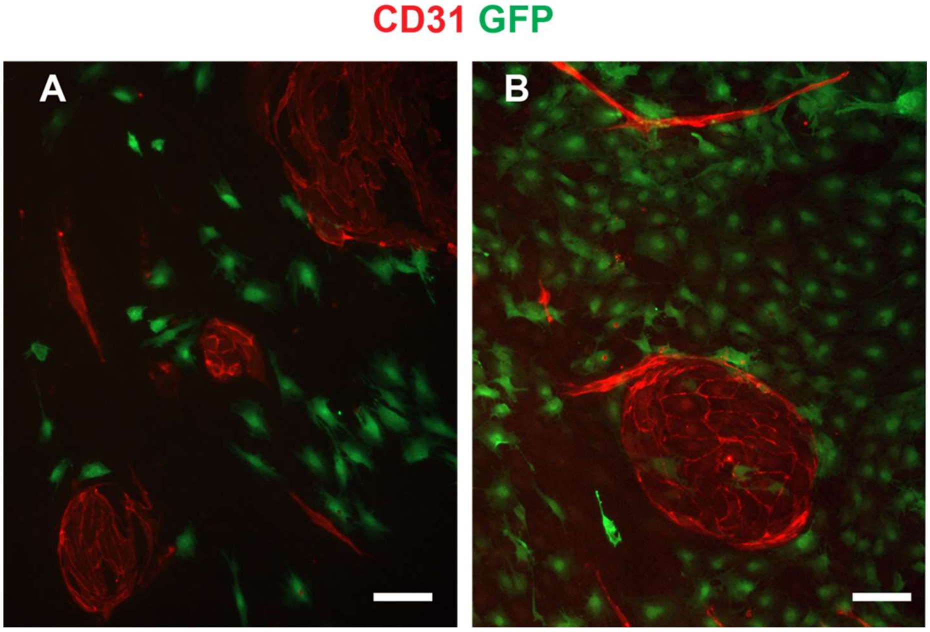

Supplementary Figure S5. GFP-MCs showed no specific alignment in co-culture with HDMECs in the absence of VEGF-A_{165}. (**A**,**B**) GFP-MCs co-seeded with HDMECs at 1:30 (**A**) or 1:1 (**B**) but without led to covering of GFP-MCs in the space around the endothelial swirls. Scale bars are 100 μm.

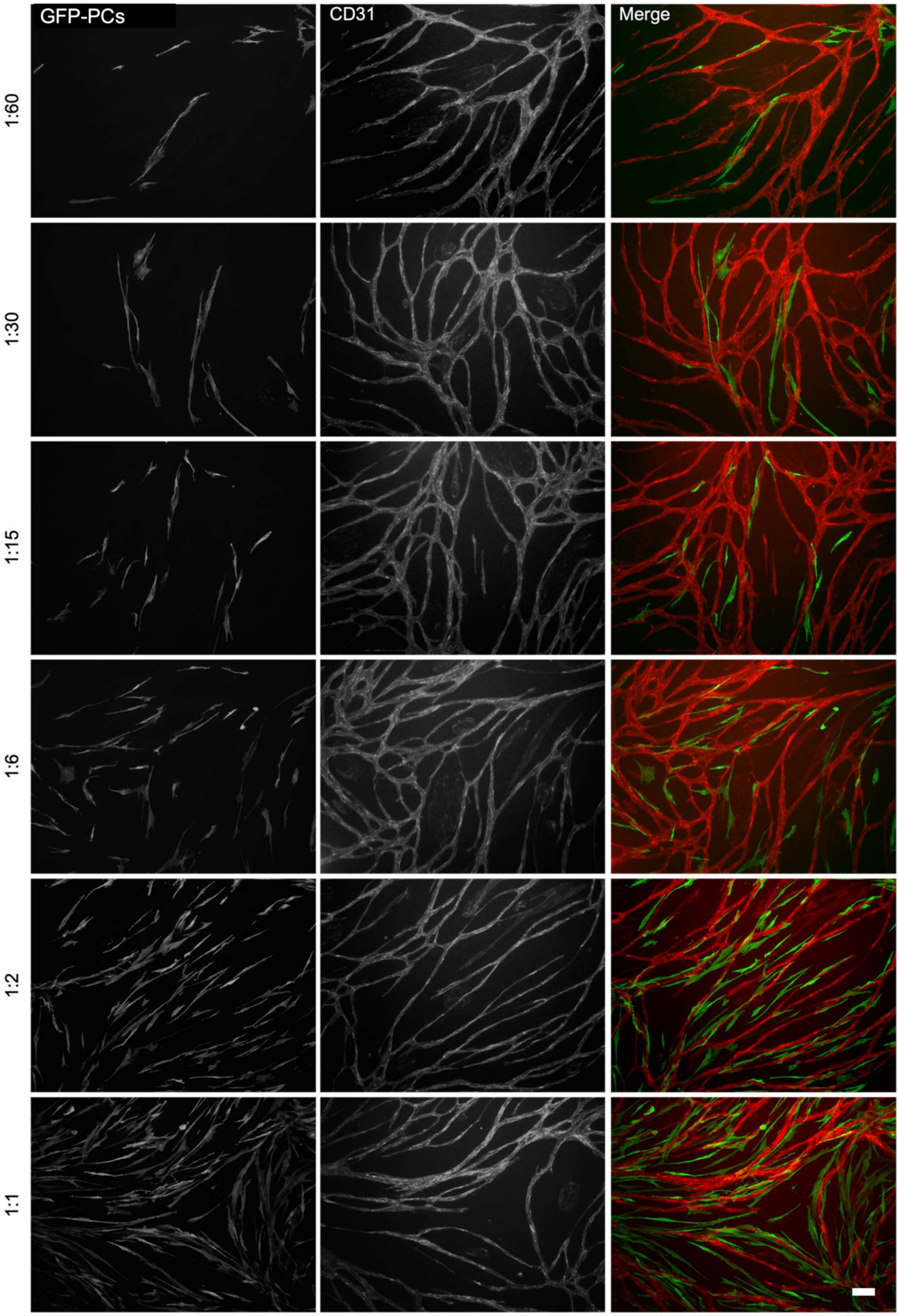

Supplementary Figure S6. Representative images of immunofluorescence staining for GFP (GFP-PCs) and CD31 showing arrangement of GFP-PCs with respect to HDMECs tubes at different seeding ratios in the vitro angiogenesis experiment in the presence of

VEGF-A165. GFP-PCs had been added into the assay at the ratios indicated (ratios shown as number of GFP-PCs : number of HDMECs). Scale bar = 100 μm.

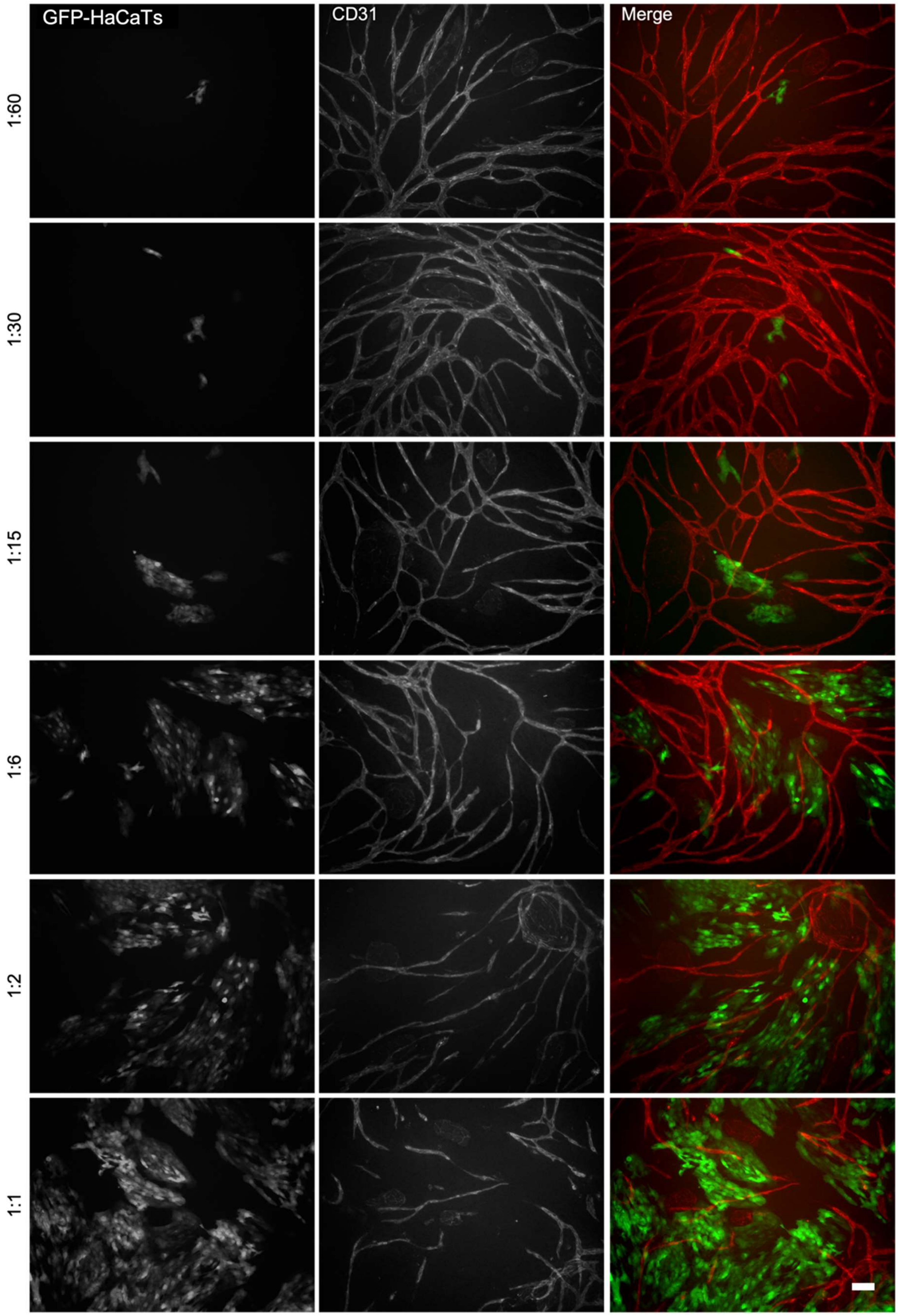

Supplementary Figure S7. Representative images of immunofluorescence staining for GFP (GFP-HaCaTs) and CD31 showing arrangement of GFP-HaCaTs with respect to HDMECs tubes at different seeding ratios in the vitro angiogenesis experiment in the presence of VEGF-A_{165}. GFP-HaCaTs had been added into the assay at the ratios indicated (ratios shown as number of GFP-HaCaTs : number of HDMECs). Scale bar = 100 μm.

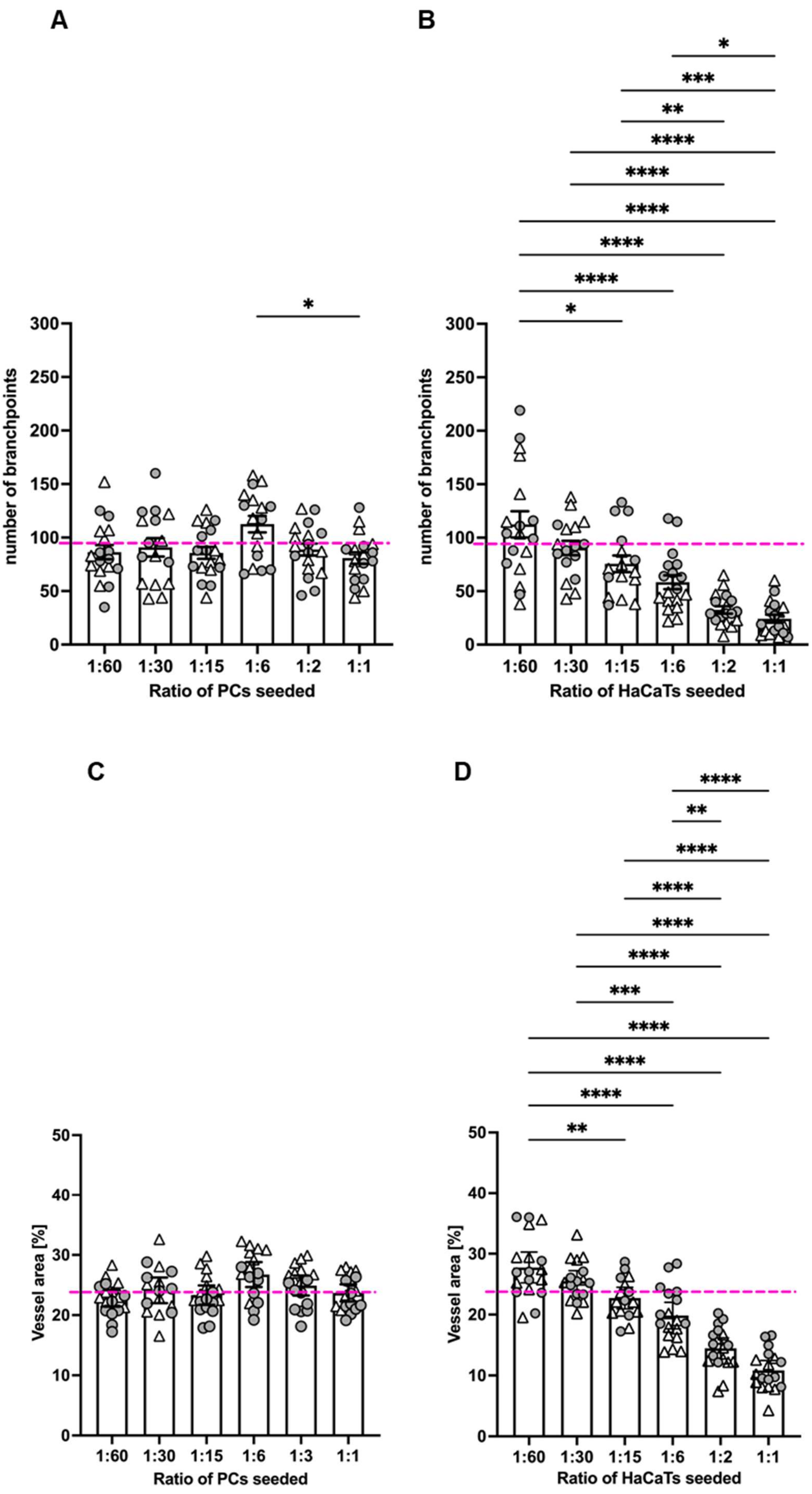
A
B
C
D
number of branchpoints
Ratio of PCs seeded
Ratio of HaCaTs seeded
Vessel area [%]
1:60
1:30
1:15
1:6
1:3
1:2
1:1

Supplementary Figure S8. Quantification of the effect on endothelial tube formation after 6 days of co-culture with Pericytes and HaCaTs. (**A**) The presence of GFP-PCs in the co-cultures had no effect on the number of branch points independently on the number of seeded GFP-PCs. There was a significant increase in branch points between when GFP-PCs were seeded at 1:6 compared to 1:1 seeding ratio. (**B**) The number of HaCaTs seeded negatively influenced the number of branch points in the co-cultures, with more cells seeded resulting in fewer branch points. (**C**) GFP-PCs in the co-cultures had no effect on the percentage vessel area. (**D**) The number of HaCaTs seeded negatively influenced the percentage vessel area in the co-cultures, we more cells seeded resulting in a smaller percentage vessel area. The pink stipled line indicates the control values for branch points and percentage vessel area in the absence of GFP-MCs, GFP-PCs or GFP-HaCaTs. Circles and triangles indicate data points from two independent experiments. Data analysed by one way ANOVA with Tukey's multiple comparison test; data shown as mean with 95% confidence interval; significance was defined by a p-value ≤ 0.05. P-value$\leq$0.05 (*), $\leq$ 0.005 (**), $\leq$0.0005 (***), $\leq$0.0001 (****).

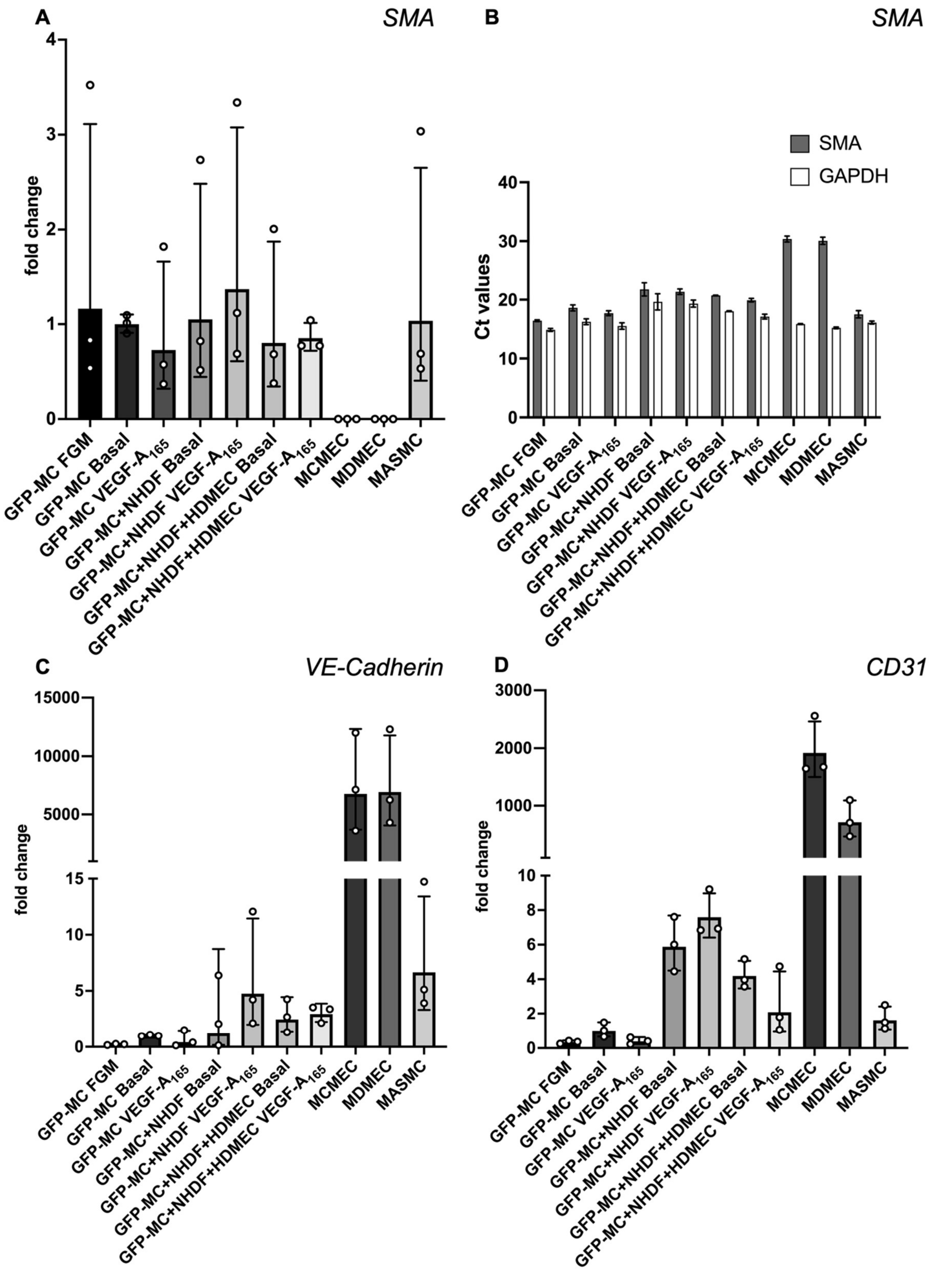

Supplementary Figure S9. Changes in gene expression of GFP-MCs in response to VEGF-A165 stimulation, co-culture with NHDF or in the angiogenesis assay. (**A**) There was no statistically significant difference in *SMA* expression for any GFP-MCs in any combination of culture when compared to GFP-MC Basal using a one-way ANOVA. (**B**) Analysis of the Ct values for *SMA* expression revealed that for all conditions (except the endothelial cells) these were almost at the same level as those for the *GAPDH* house keeping gene. (**C**,**D**) Expression of *VE-Cadherin* (**C**) and *CD31* (**D**) was very high compared to GFP-MCs under

all the other conditions or in the mouse primary arterial smooth muscle cells (MASMCs). FGM - full growth media, MCMEC – mouse primary cardiac microvascular endothelial cells, MDMEC – mouse dermal microvascular endothelial cells.

Data displayed as Geometric mean with geometric SD.

Supplementary Table S1. Human primer pairs used in the qRT-PCR.

	**Human primers**	
	**forward (5'-3')**	**reverse (5'-3')**
-ACTIN	GATGAGATTGGCATGGCTTT	CACCTTCACCGTTCCAGTTT
CD31	CAGGAGCACCTCCAGCCAACTT	ATCCTGGGCTGGGAGAGCATTTCG
VEGFR2	CAAACGCTGACATGTACGGTCT	CCAACTGCCAATACCAGTGGA
VEGFR3 (FLT4)	GCTCCTACGTGTTCGTGAGAGA	TCCTGTTGACCAAGAGCGTG
PODOPLANIN	CTGCTCTTCGTTTTGGGAAG	GGTTCCTGGAGTCACCACAT
PROX1	CAAAAATGGTGGCACGGAG	CCTGATGTACTTCGGAGCCTGT

Supplementary Table S2. Mouse primer pairs used in the qRT-PCR.

	**Mouse primers**	
	**forward (5'-3')**	**reverse (5'-3')**
CD31	AGCCTAGTGTGGAAGCCAAC	TCGACCTTCCGGATCTCACT
Ng2	GCATCATCATTCCGGTGTGC	GGTCAACACCTGGACATCGT
SMA	AGGCCGGCTTCGCTGGTGAT	TCTGGGCTTCATCCCCCACA
Pdgfrβ	GTTAGTTTTGTCTACTCGCAAC	TCATCGTCCTCGTGGAAGG
Zeb1	GGGGAAACCGCAAGTTCAAG	AGCCAGAATGGGAAAACCGT
VE-cadherin	ATTGGCCTGTGTTTTCGCAC	CACAGTGGGGTCATCTGCAT
Msln	TGTTCCGGCAGCATCAGAAA	GGCCTCTCGGACATTGAAGT
GAPDH	ACCAGGGCTGCCATTTGCAG	TCGGCCTTGACTGTGCCGTT

Supplementary Table S3. Mean values for area, perimeter and circularity and 95% confidence intervals.

	GFP-MC BASAL	GFP-MC VEGF-A_{165}	NHDF+GFP-MC BASAL	NHDF+GFP-MC VEGF-A_{165}
**AREA [m^2]**	**668.3**	**926.8**	**1331**	**1273**
Lower 95% CI	644.2	889.1	1281	1220
Upper 95% CI	692.4	964.6	1382	1326
**PERIMETER [m]**	**101.8**	**120.1**	**152.2**	**149.2**
Lower 95% CI	100	117.6	149.2	145.8
Upper 95% CI	103.6	122.7	155.2	152.5
**CIRCULARITY**	**0.7702**	**0.7517**	**0.6898**	**0.6838**
Lower 95% CI	0.765	0.7461	0.6832	0.6763
Upper 95% CI	0.7753	0.7573	0.6964	0.6913

Supplementary Movie S1:

Movie showing mesothelial cell interaction with endothelial cells during network formation.

Deposited on Zenodo, DOI: https://doi.org/10.5281/zenodo.8238365
